# Evaluation of a New Topical Treatment for the Control of Cutaneous Leishmaniasis

**DOI:** 10.3390/microorganisms8111803

**Published:** 2020-11-17

**Authors:** Berenice Martínez-Salazar, Vanessa Carregaro Pereira, Yazmin Hauyon-La-Torre, Ali Khamesipour, Fabienne Tacchini-Cottier

**Affiliations:** 1 Department of Biochemistry, University of Lausanne, 1066 Epalinges, Switzerland; maria.martinezsalazar@unil.ch (B.M.-S.); vcarregaro@usp.br (V.C.P.); yazmin.hauyon@unil.ch (Y.H.-L.-T.); 2 Centre for Research and Training in Skin Diseases and Leprosy, Tehran University of Medical Sciences, Tehran 14166, Iran; ali.khamesipour@gmail.com

**Keywords:** *Leishmania*, *L. major*, topical treatment, MF-29, anti-leishmanial drug

## Abstract

*Leishmania major* (*L. major*) causes cutaneous leishmaniasis in the Old World. The infection mostly induces a localized lesion restricted to the sand fly bite. The costs and the side effects of current treatments render imperative the development of new therapies that are affordable and easy to administrate. Topical treatment would be the ideal option for the treatment of cutaneous leishmaniasis. MF29 is a 3-haloacetamidobenzoate that was shown in vitro to inhibit tubulin assembly in *Leishmania*. Here, we tested a topical cream formulated with MF29. BALB/c mice were infected in the ear dermis with *L. major* metacyclic promastigotes and once the lesion appeared, mice were treated with different concentrations of MF29 and compared to the control group treated with the cream used as the vehicle. We observed that topical application of MF29 reduced the progression of the infection while control groups developed an unhealing lesion that became necrotic. The treatment decreased the type 2 immune response. Comparison with SinaAmphoLeish, another topical treatment, revealed that MF29 treatment once a day was sufficient to control lesion development, while application SinaAmphoLeish needed applications twice daily. Collectively, our data suggest that MF-29 topical application could be a promising topical treatment for cutaneous leishmaniasis.

## 1. Introduction

Leishmaniasis is a neglected tropical disease that is a major public health issue. It predominantly affects people of low socioeconomic status. It is the second most deadly vector transmitted disease with over 1 billion people living in endemic areas and at risk of infection according to the World Health Organization. More than 20 *Leishmania* species infect humans, they are transmitted during the blood meal of female phlebotomine sandflies. Infection can cause a wide spectrum of diseases including visceral (VL), cutaneous (CL), and mucocutaneous (MCL) forms. The most predominant form of leishmaniasis is CL, with an estimated global incidence of 600,000 to one million cases each year [1]. Upon infection, CL leads to the formation of a papule that develops into an ulcer at the site of the insect bite. Healing may be long (3–18 months) depending mostly on the infecting *Leishmania* species and the host immune status. Although not fatal, CL has profound socio-economic impacts due to the stigmatization of infected and cured individuals, as these latter may bear disfiguring scars [2,3]. Despite this, most of the current efforts in drug development are focused on VL and drug development against CL lags behind [4]. Current WHO recommendations for CL treatment depend on the country where the patient is infected, the clinical form of the disease, and the infecting *Leishmania* spp., moreover current treatments show various degrees of efficacy (reviewed in [5]). Local therapies are recommended for infection due to *L. major* and *L. mexicana*, as well as for other *Leishmania* spp. that give rise to small or a low number of lesions. To this end, there has been a growing interest in developing safe, affordable, non-parenteral efficacious topical treatments.

In light of this, the use of experimental models of infection are very informative. For instance, infection of inbred BALB/c mice with a high dose of *L. major* has long been used to test drug efficacy of potential anti-leishmanial compounds in vivo [6]. Infection in these mice induces the development of nonhealing lesions linked with the development of a Th2 immune response whereby the mice fail to control parasite load. In contrast, infection with *L. major* in C57BL/6 mice induces self-healing lesions correlating with the development of a Th1 immune response and control of parasite load [7]. However, the development of a T helper response does not always correlate with the outcome of infection [8,9]. For example, infection of C57BL/6 mice with *L. major* Seidman (Sd), a strain derived from a patient with a nonhealing lesion, induces the development of progressive lesions despite the presence of a Th1 immune response, providing an alternative model closer to nonhealing CL observed in humans [10].

In the present study, we used experimental murine models of *L. major* infection to investigate the impact of topical administration of the anti-microtubule drug MF29, an ethyl 3-chloroacetamidobenzoate, on *L. major*-induced cutaneous lesion development. Microtubules are involved in many processes implicated in motility, maintenance of cell shape, cell transport, and cell division [11]. The MF29 anti-microtubule drug was previously shown to display some anti-*Leishmania* effects in vitro and also in vivo when injected intra-peritoneally in *L. major* infected BALB/c mice [12]. Here, using in vivo mouse models, we aimed to investigate the efficacy of an MF29 formulated cream in topical applications following infection with *L. major* LV39 in BALB/c mice. We also tested the MF29 topical formulation following *L. major* Sd infection in C57BL/6 mice. In each model, daily topical treatment with MF29 upon lesion appearance resulted in a significant beneficial impact on the control of pathology, suggesting a promising use for this compound in topical treatments.

## 2. Materials and Methods

### 2.1. Mice and Ethics

BALB/c and C57BL/6 mice were purchased from Envigo (Cambridgeshire, United Kingdom) and bred under specific pathogen-free conditions at the animal facility unit of the University of Lausanne, Epalinges. Six- to eight-week-old mice were used for the experiments. Animal experimentation protocols were approved by the veterinary office of the Canton of Vaud (Authorization 1266.6-7 to F.T.C.) and were undertaken in accordance with cantonal and federal laws as well as the ethical principles of the Basel declaration.

### 2.2. Parasites and Infections

All the parasites were passed through BALB/c mice to keep the virulence of the strains. *Leishmania major* LV39 (MRHO/Sv/59/P) and *L. major* LV39-mCherry [13] were cultured in rabbit agar blood in 10 mL of supplemented M199 media with 5% heat-inactivated fetal calf serum. *L. major* Seidman (MHOM/SN/74/SD), *L. major* Sd-RFP [14] and *L. mexicana* (MNYC/BZ/62/M379) were cultured in M199 media supplemented with 10% heat-inactivated fetal calf serum. All the cultures were grown at 26 °C for 5 passages maximum. For the infection of mice, metacyclic promastigotes were isolated as previously described [15]. 10^5^ metacyclic promastigote parasites were inoculated into the ear dermis. The lesion development was analyzed and scored as previously described [16]. Briefly, lesion score ranges from 0 to 8. This scoring system takes into account the first signs of inflammation (score 0.5) to palpable swelling (score 1). As soon as lesion borders are defined, the lesion dimension (lesion length and width) is measured with a caliper and the longest dimension (mm) is used to assign a score with 0.5 mm increments. The first signs of necrosis are given a score of 6, while necrosis severity increases the scores to 7–8.

### 2.3. Drugs and Treatments

The ethyl 3-(2-chloroacetamido) benzoate, named MF29, is an ethyl benzoate derivative of 3-haloacetamidobenzoic acids (CAS No is 58915-19-8). Its formula is C_11_H_12_ClNO_3_, its molecular weight is of 241.67 and its lipophilicity (LogP) is 2.07. The MF29 structure has been previously published [12]. The MF29 compound was reconstituted with 1 mM dimethyl sulfoxide (DMSO, Sigma) for the in vitro studies. For the in vivo studies, it was formulated in absence of DMSO as previously described [12]. For the 1, 0.5, 0.25 and 0.125 μM concentrations, serial dilutions were performed in DMEM media supplemented with 5% heat-inactivated FCS. The stock solution was kept at 4 °C and fresh serial dilutions were made for every experiment. For in vivo experiments, the cream vehicle and the MF29 topical cream at 1% were produced by BioNoox and were kept at 4 °C. The vehicle cream contains regular cosmetically acceptable excipients and is proprietary of BioNOOX SA. Once the lesion reached a score of 2 (visible lesion), after an average time of 12 days post-infection, the topical treatments were applied once or twice a day, as indicated. Mice were euthanized and analyzed 24 h after the final topical application as indicated. The formulated cream (10 μL) was applied with an insulin syringe tip to cover the lesion. Following the state of Vaud veterinary rules, all the treatments were stopped, and mice were euthanized upon signs of necrosis.

### 2.4. Murine Neutrophil Isolation and Macrophage Differentiation

Bone marrow from the femora and tibia of either BALB/c or C57BL/6 mice were flushed with RPMI. Erythrocytes were lysed with ACK buffer, the remaining leukocytes were washed with MACS buffer and the neutrophils were isolated by positive MACS using the neutrophil isolation kit (Miltenyi Biotec) according to the manufacturer’s indications. The negative selection was cultured for 7 days to differentiate into bone marrow-derived macrophages (BMDM). To this end, the cells were cultured in DMEM supplemented with 5% fetal calf serum, 20% L929 (M-CSF differentiation factor), and 5% penicillin/streptomycin.

### 2.5. Parasite Viability, IC_50_ and EC_50_ Determination

The IC_50_ is defined as the drug concentration causing 50% inhibition of proliferation with respect to untreated parasites. Promastigotes from the stationary phase were washed twice with non-supplemented M199 and centrifuged for 10 min at room temperature. The pellet was then resuspended in supplemented M199 medium, the parasites were counted in a Neubauer chamber and the number was adjusted to 3 × 10^6^/200 μL. The MF29 was added at different concentrations ranging from 4 to 0.125 μM. After 8 h of incubation at 26 °C, Alamar blue was added to the samples at a 1× final concentration and incubated for 12 h following the manufacturer’s instructions. Viability was subsequently measured by fluorescence in the Spectra Max I3 multimode microplate reader and reported as relative fluorescence units. The IC_50_ values were determined by non-linear regression using Prism GraphPad 10. The EC_50_, the 50% of the effective concentration to induce maximal killing of the parasites in infected cells, was measured by FACS. Bone marrow-derived macrophages were infected with fluorescent parasites: *L. major* mCherry, Lm-Sd-RFP, and *L. mexicana* DsRed. After 4 h, non-phagocytosed parasites were washed by pipetting PBS in the plate at room temperature. The drugs were then added at a concentration of 0.5, 0.25 and 0.125 μM in supplemented DMEM, and a group without drugs was kept as control. The cells were cultured for 48 and 96 h and then harvested with cold dissociation buffer (1× PBS with 2% EDTA). The harvested cells were washed with FACS buffer (1× PBS with 2% FCS), acquired in a BD-LSRII Fortessa and results were analyzed by FlowJo 10. The EC_50_ was determined with a non-lineal regression using Prism GraphPad 10.

### 2.6. Tissue Processing

Ears and draining lymph node cells were recovered and processed into a single-cell suspension as previously described [15]. Briefly, tissues were digested in Liberase (Roche) for 2 h at 37 °C and dLN homogenized before further processing in 40 µm filters to obtain a single-cell suspension.

### 2.7. Flow Cytometry Analysis

The following mAbs were used for cell surface staining: anti-CD45-PerCpCy5.5, anti-Ly6G(1A8)-PE or FITC, anti-Ly6C-APC or FITC (all from BD Bioscience), anti-CD11b-Pacific blue, anti-CD11c-PECy7, anti-CD4-AF700, anti-IL-4-FITC, and anti-IFN-γ-PECy7 (all from eBioscience); anti-CD3-PE or APC and anti-IL-10-PE (Biolegend); and anti-CD8-APC (Invitrogen). For the exclusion of dead cells, we used the Live/Dead fixable Aqua Dead Cell Stain Kit (Invitrogen). *L. major* mCherry was detected using Texas red as a positive control, *L. mexicana* DsRed and *L. major* Sd-RFP were detected using PE as a positive control. Fluorescent parasites and stained murine cells were acquired using a flow cytometry analyzer, either BD LSRII or BD LSR-Fortessa Series (Beckton Dickinson) machines, the data was acquired with the DIVA software and the results were analyzed with FlowJo 10 software.

### 2.8. Determination of the IgG Antibody Response

The levels of *L. major*-specific IgG1 and IgG2b antibodies were determined by ELISA in the sera of infected BALB/c mice as previously described [17]. Sera were obtained 40 days post infection at the end of the experiment. Biotinylated goat anti-mouse IgG2 (Southern Biotech) and biotinylated rat anti-mouse IgG1 (BD Pharmingen) were used. Plates were read at an optical density of 490 nm. A titration curve was performed for all samples.

### 2.9. Parasitic Load Determination

Bone marrow-derived macrophages (BMDM) were harvested with cold PBS, washed with DMEM, and counted. The cells were plated in a 24 well plate, 1 × 10^6^ cells were used per condition. The macrophages were plated and left to attach to the plate for two hours at 37 °C. Once attached, the parasites were added to a multiplicity of infection (MOI) 10. The cells were then centrifuged at 1500 rpm, 10 min at room temperature and kept at 26 °C for 4 h to allow the parasites to infect the macrophages. After 4 h, the non-phagocytosed parasites were washed by gentle pipetting with PBS at room temperature. Complete DMEM and 1 to 0.125 µM MF29 was then added to the cells. The cells were harvested after 4, 48, and 72 h of culture, washed with PBS, and resuspended in FACS buffer. The cells were acquired in the BD LSR II-Fortessa machine, and the results analyzed using FlowJo 10. Parasite load in infected organs was determined by limiting dilution assay as previously described [13].

### 2.10. Statistics

For comparison between two sample groups with a normal distribution, statistics were assessed by a non-parametric unpaired *T*-test (Mann-Whitney *U*-test). Comparisons of more than two groups were made by one-way ANOVA and Bonferroni post-hoc test. * *p* < 0.05, ** *p* < 0.005, *** *p* < 0.0002, **** *p* < 0.0001, ns = not significant.

## 3. Results

### 3.1. In Vitro Anti-Leishmanial Activity of MF29

#### 3.1.1. MF29 Anti-Leishmanial Activity

**Figure 1:**
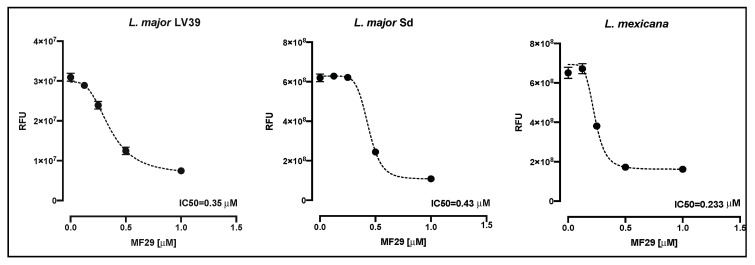
Effect of the MF29 compound against *Leishmania species*. Metacyclic promastigotes were cultured for 8 h in the presence of MF29 at 1, 0.5, 0.25, and 0.125 μM concentrations, and the IC_50_ was assessed by Alamar blue. The figure is representative of one out of three experiments. Each point represents the mean ± SEM (*n* = 3 per group).

MF29 has been shown to have a detrimental effect on *Leishmania* microtubule organization, with an impact on parasite survival [12]. Promastigote susceptibility to MF29 was determined, testing MF29 concentrations between 1 to 0.125 μM against *L. mexicana* and two distinct strains of *L. major* that all cause cutaneous leishmaniasis (Figure 1). *L. major* LV39, as for many other *L. major* strains, leads to the development of a small lesion that is self-healing upon infection of C57BL/6 mice. In contrast, *L. major* Sd that was isolated from the non-healing lesion of a patient resistant to drugs [10] leads to the development of a progressive non-healing lesion upon infection in C57BL/6 mice. Exposure of *Leishmania* promastigotes in vitro to MF29 had a negative impact on the two *L. major* strains and the *L. mexicana* spp. analyzed, with IC_50_ values below 0.5 µM.

#### 3.1.2. Anti-Leishmanial Activity of MF29 on Infected Macrophages

**Figure 2:**
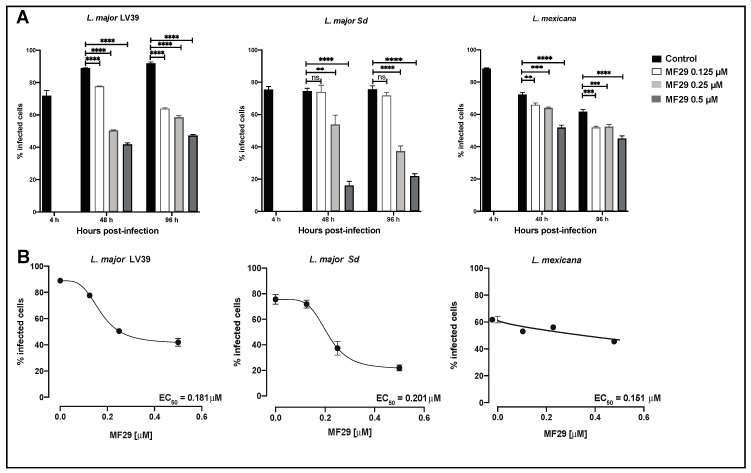
In vitro activity of MF29 against *Leishmania*-infected cells and EC_50_ assessment. (**A**) BMDM were infected at a MOI 10 with *L. major* LV39, *L. major* Sd, and *L. mexicana*. As a control, another infection was performed for 4 h without further stimulation. The infected cells were incubated in presence of MF29 at a 0.5, 0.25, and 0.125 µM concentration and at the time points of 48, and 96 h and the frequency of infected cells was analyzed (**B**). The EC_50_ was determined with a non-linear regression by measuring the parasitic load at 96 h post-infection, in *L. major* LV39, *L. major* Sd, and *L*. *mexicana*-infected cells. Data are represented as mean ± SEM, *n* = 3 per group Two-Way ANOVA (Tukey’s post-hoc test). ** *p* < 0.001, *** *p* < 0.0002, **** *p* < 0.0001, ns = not significant.

After having shown that MF29 acts on *Leishmania* promastigote parasites in culture, we aimed to assess the effect of MF29 on the replicating amastigote form of the parasite residing in infected macrophages. BMDMs were infected with fluorescent *Leishmania* parasites at a MOI of 10. After 4 h of infection, the cells were washed and exposed to different concentrations of MF29 ranging from 0.5, 0.25 to 0.125 μM. Non-exposed infected cells were maintained as controls. The frequency of infected macrophages was analyzed after 48 and 96 h by flow cytometry (Figure 2A). We found that a concentration of 0.125 μM of MF29 already induced a small frequency of *L. major* LV39 and *L. mexicana* intracellular killing. An effect on *L. major* Sd infected macrophages was observed only with higher concentrations of 0.25 and 0.5 μM (Figure 2A). At a concentration of 0.25 μM, MF29 reduced the infection in half of the *L. major* LV39 infected cells at both 48- and 96-h post-infection. Increasing MF29 concentration to 0.5 μM of MF29 had no further impact on parasite killing for *L. major* LV39. MF29 showed the most significant effect on macrophages infected with *L. major* Sd when treated with 0.5 μM MF29, with a drop of infection frequency by 60 points. MF29 also induced *L. mexicana* amastigote killing which increased at 0.5 μM concentration (Figure 2A). The data obtained 96 h after infection were used to determine the EC_50_ (Figure 2B). We found that the *L. major* Sd strain had the highest EC_50_ values, followed by *L. major* LV39 and *L. mexicana* (Figure 2B). Collectively, we show here that MF29 at a concentration of 0.25 μM is able to induce the intracellular killing of the three *Leishmania* tested.

### 3.2. In Vivo Topical Treatment of Mice Infected with L. major

#### 3.2.1. BALB/c Mice Treated Once a Day with MF29 Cream Better Control Disease Development

**Figure 3:**
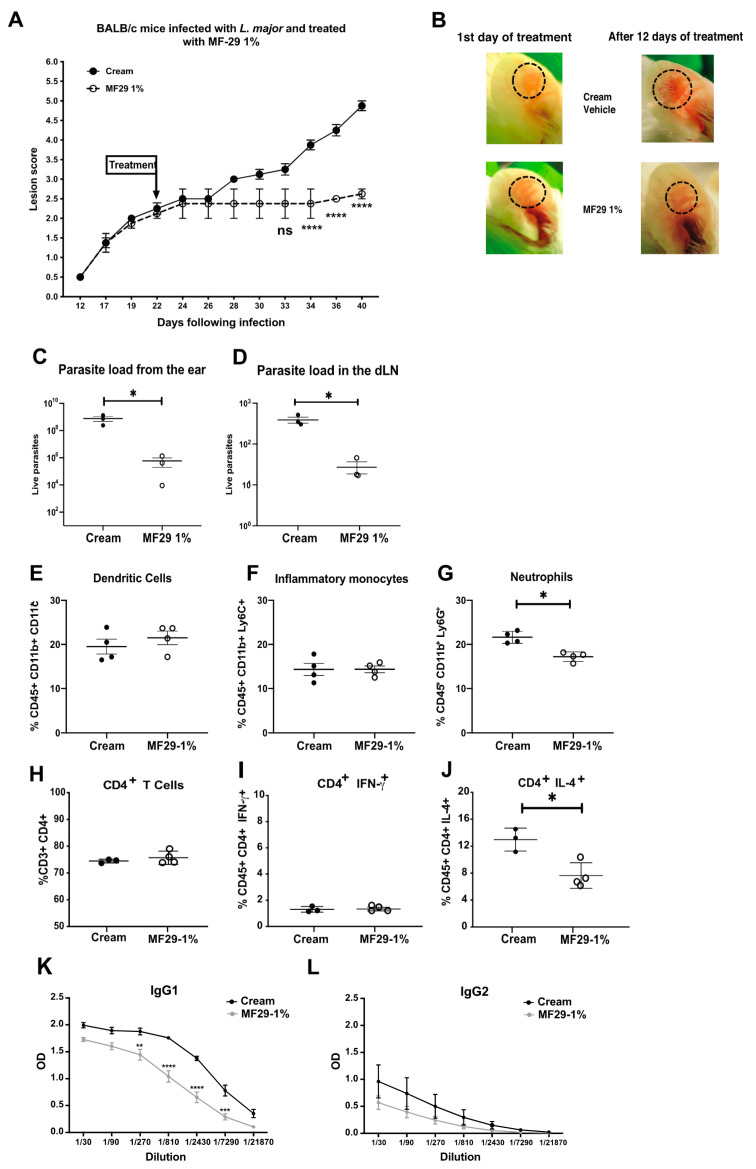
Topical treatment with MF29 1% decreases cutaneous pathology. BALB/c mice were infected i.d. with metacyclic *L. major* LV39 promastigotes and ears were treated once daily with either vehicle cream or with MF29 1% cream once the lesion score reached 2. (**A**) Lesion score following infection. Data are mean ± SEM, Two-Way ANOVA (Bonferroni’s post-hoc test) * *p* < 0.05, **** *p* < 0.0001, ns = not significant. (**B**) Representative pictures from an ear at the onset and after 12 days of treatment with vehicle cream and MF29 1% topical cream. (**C**) Forty days post-infection, the parasite load was determined in infected ears and (**D**) draining lymph nodes. (**E**) The frequency of monocyte-derived dendritic cells, (**F**) inflammatory monocytes, and (**G**) neutrophils was also determined. The frequency of dLN (**H**) CD4^+^ T cells, (**I**) CD4^+^ INF-γ^+^ and (**J**) CD4^+^IL-4^+^ was determined by flow cytometry. The data are represented as mean ± SEM, non-parametric *T*-test (Mann–Whitney *U*-test), (**K**) the relative amount of *L. major*-specific IgG1 and of (**L**) IgG2b present in the sera of mice was analyzed by ELISA, Data are mean ± SEM, Two-Way ANOVA (Bonferroni’s post-hoc test) ** *p* < 0.005, *** *p* < 0.0005, **** *p* < 0.0001. The data are representative of three experiments with ≥4 mice/group. * *p* < 0.05.

As we demonstrated the impact of MF29 in infected macrophages in vitro, we wanted to investigate if a cream containing MF29 could be effective as a topical treatment for cutaneous leishmaniasis in vivo. MF29 cream was formulated at different concentrations (0.1%, 0.5%, and 1%). We infected BALB/c mice i.d. with 10^5^ metacyclic *L. major* LV39 promastigotes. Once the lesion was visible and reached a score of 2, the treatment with the MF29 0.1, 0.5 or 1% was applied once daily at the same time of the day and the mice were euthanized as indicated 24 h after the last day of treatment. We did not find any effect on lesion development when applying MF29 at a concentration of 0.1 and 0.5% (data not shown). However, mice treated with the MF29 1% cream developed significantly smaller lesions than the control group (Figure 3A,B). The experiment was ended 40 days post-infection when the first signs of necrosis appeared in the control group, and 24 h after the last topical application. The infected ears and draining lymph nodes were subsequently isolated from euthanized mice and the parasite load was analyzed by LDA. A significant decrease in parasite burden was observed in the ears (Figure 3C) as well as in the dLN (Figure 3D) of mice treated with MF29 1% compared with those obtained in the same organs of the control group of mice. There was no difference in the frequency of dendritic cells (Figure 1E), or inflammatory monocytes (Figure 1F) recruited to the infected ear but there was a decreased proportion of neutrophils (Figure 1G) in the mice treated with the MF29 1% cream, in line with the lower inflammation observed in the MF29 1% treated mice.

We then investigated whether the treatment with MF29 1% had an impact on the adaptive immune response. In this well-established experimental model, infection of BALB/c mice with *L. major* induces a Th2 immune response characterized by high levels of CD4^+^ Th2 cells producing IL-4 and low levels of CD4^+^ IFN-γ^+^ Th1 cells (reviewed in [7]). BALB/c mice were infected with *L. major* LV39 and topical treatment with MF29 1% or control vehicle cream was initiated as indicated in Figure 3A, at a time when the lesion was well defined (score 2). Treatment was applied daily and 40 days post infection, one day after the last topical application, mice were euthanized and tissue cells analyzed. We found that the proportion of CD4^+^ T cells in dLNs was similar between both groups (Figure 3H). The frequency of IFN-γ production by CD4^+^ T cells observed was low and similar in dLN cells of mice treated, or not, with MF29 1% (Figure 3I). In contrast, the frequency of CD4^+^IL-4^+^ T cells was reduced in the group of mice treated with MF29 1% (Figure 3J). Accordingly, the IL-4 and IL-13-dependent IgG1 levels decreased in the serum of mice treated with MF29 1% (Figure 3K) compared to the serum of mice treated with vehicle cream. In contrast, IgM/D switching to IgG2b levels, a process that depends on IFNγ, was low and at similar levels between mice treated with vehicle or MF29 1% cream (Figure 3L). Very low frequencies (0.5–1%) of CD4^+^IL-10^+^ producing cells were observed with similar frequencies in dLN cell of mice treated or not with MF29 1% (data not shown). Collectively, these results show that topical treatment with MF29 1% decreases significantly the development of the inflammatory lesion and the frequency of CD4^+^ Th2 cells.

#### 3.2.2. Increasing the Frequency of MF29 Topical Treatment Does Not Modify Improved Lesion Control

**Figure 4:**
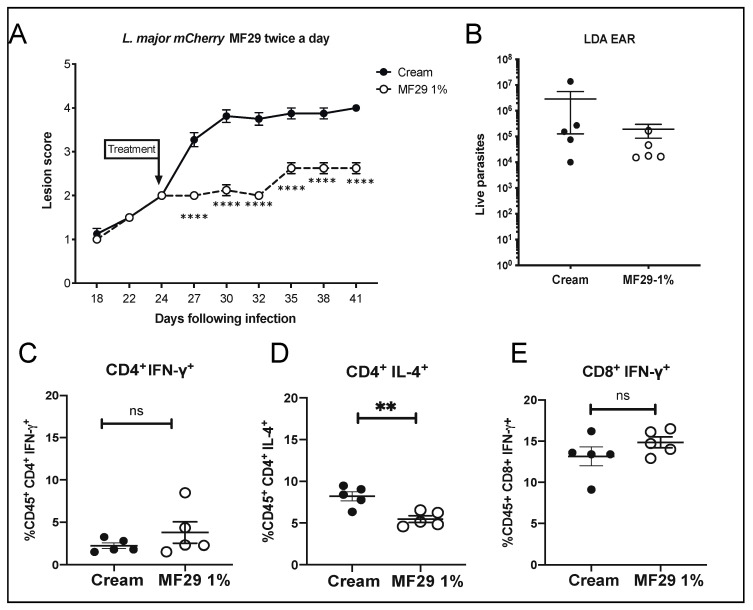
Topical application of MF29 1% twice per day. BALB/c mice were infected i.d. with metacyclic *L. major* LV39 promastigotes and ears were either treated twice daily with vehicle cream or MF29 1% cream once the lesion score reached 2. (**A**) Lesion development, data are mean ± SEM, Two-Way ANOVA (Bonferroni’s post-hoc test) ** *p* < 0.005, ns = not significant. (**B**) Forty-one days after infection, the parasite load in the infected ear was determined by limiting dilution assay (LDA). (**C**) The frequency of CD4^+^INF-γ^+^, (**D**) CD4^+^IL-4^+^ and (**E**) CD8^+^IFN-γ^+^ was determined by flow cytometry in the dLN. Data are represented as mean ± SEM, non-parametric *T*-test (Mann–Whitney *U*-test) ** *p* < 0.07, ns = not significant. The data are representative of two experiments including 5 mice/group.

We showed that topical treatment with MF29 1% applied once a day had a significant impact on the progression of the cutaneous lesion. Therefore, we investigated whether similar treatment applied twice a day could increase the effect on lesion development and parasite control. We infected mice i.d. with 10^5^ metacyclic parasites and treated them twice a day once the lesion reached a score of 2. Mice similarly treated with cream vehicle were used as a control group. We observed again that MF29 1% treatment had a major impact on lesion development, decreasing significantly lesion score (Figure 4A). Parasite load at the site of infection was lower than the control group but this was not statistically significant in this experiment (Figure 4B). We again found only a slight effect of MF29 treatment on IFN-γ production in CD4^+^ (Figure 4C) and CD8^+^ T cells (Figure 4E). A significant decrease in the frequency of IL-4 producing CD4^+^ T cells was again found in the MF29 1% treated group (Figure 4D). Thus, MF29 1% applied twice a day has an impact on lesion development and IL-4 production by CD4^+^ T-cells, together with a small impact on the parasitic load. However, increasing the frequency of MF29 1% treatment twice a day did not ameliorate its impact on pathology.

### 3.3. Topical Treatment with MF29 Following Infection with L. major Seidman Induces Better Lesion Control

**Figure 5:**
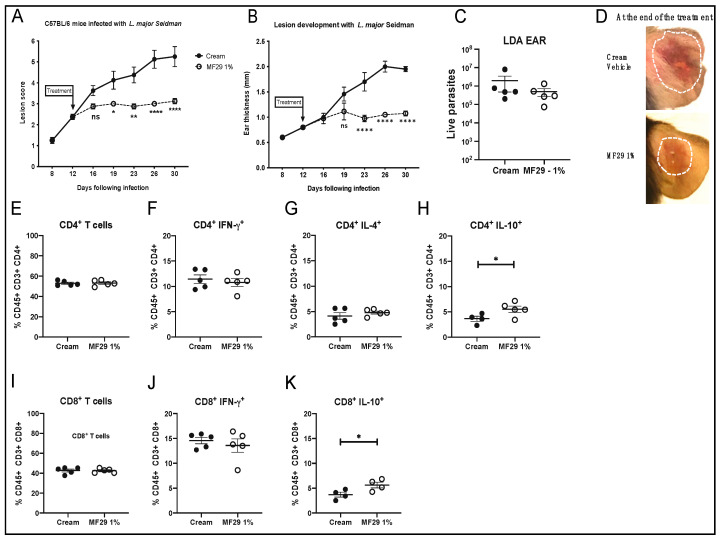
Topical treatment with MF29 1% significantly decreases cutaneous pathology following infection with *L. major* Seidman (Sd). C57BL/6 mice were infected i.d. with metacyclic *L. major* Sd promastigotes. Topical treatment with vehicle cream (cream) or MF29 1% cream was initiated once daily as indicated. The lesion development is shown as (**A**) lesion score and (**B**) ear thickness. Data are represented as mean ± SEM, Two-Way ANOVA (Bonferroni’s post-hoc test) * *p* < 0.05, ** *p* < 0.002, **** *p* < 0.0001, ns = not significant. (**C**) Parasite load in the infected ears was determined by LDA. (**D**) Representative picture of one ear, either treated with vehicle, or with MF29 1% cream. (**E**) The frequency of dLN CD4^+^ T cells, (**F**) CD4^+^ IFN-γ^+^, (**G**) CD4^+^IL-4^+^, (**H**) CD4^+^IL-10^+^, (**I**) CD8^+^ T cells and (**J**) CD8^+^ IFN-γ^+^ and (**K**) CD8^+^ IL-10^+^ is shown. Data are represented as mean ± SEM, non-parametric *T*-test (Mann–Whitney U-test). They are representative of two experiments with ≥4 mice/group. * *p* < 0.05, ns = not significant.

Since disease control was observed in mice treated topically with MF29 1% once a day, we wanted to investigate whether similar treatment would have an impact following infection with *L. major* Sd. Infection with this *L. major* strain leads to the development of a non-healing lesion in C57BL/6 mice, a process that occurs despite the strong presence of IFN-γ producing CD4^+^Th1 cells [10]. We infected C57BL/6 mice in the ear dermis with 10^5^ *L. major* Sd promastigotes. Following our established protocol, once the lesion size reached a score of 2, the treatment with MF29 1% was applied once daily. We followed the evolution of lesion development and observed a significantly reduced lesion score in the ears of the mice treated with MF29 1% (Figure 5A). These results correlated with a significant reduction of lesion thickness in ears treated with MF29 1% with a lesion thickness maintained at around 1 mm over the infection time, while the control group showed the double of ear thickness lesion at that time of infection (Figure 5B). The mice were euthanized when the ear dermis from the mice treated with the cream vehicle showed severe injury with the onset of necrosis. Necrosis was not observed in mice treated with MF29 1% (Figure 5D). At the time analyzed, the treatment induced a small decrease in the parasitic load in treated groups, but this was not statistically significant (Figure 5C). We did not find differences in the frequency of CD4^+^ (Figure 5E) or CD8^+^ (Figure 5I) T cells. The frequency of IFN-γ producing CD4^+^ T cells (Figure 5F) and IFN-γ producing CD8^+^ T cells was high, but similar in both groups (Figure 5J). The low frequency of CD4^+^IL-4^+^ T cells observed in dLN cells was also similar in the mice treated with MF29 1% or control vehicle cream (Figure 5G). Previous reports showed that the absence of IL-10 is detrimental for the healing of *Lm*-Sd infection in C57BL/6 mice [18]. A very small increase in IL-10 production was found in both CD4^+^ (Figure 5H) and CD8^+^ (Figure 5K) dLN T cells in mice treated with MF29 1%. Collectively, the data show that following *L. major* Sd, MF29 topical treatment does not modify the frequency of IL-4 and IFN-γ production by CD4^+^ dLN T cells. IL-10 production is linked to pathology in this model of infection [18]. However, despite a small increase in the frequency of IL-10 producing-T cells observed following topical treatment with MF29 1%, the lesion healed significantly better in mice treated with MF29 1% than in the control group.

### 3.4. Comparison of MF29 and Sina-Ampholeish Topical Treatment on L. major LV39 Infected Mice

**Figure 6:**
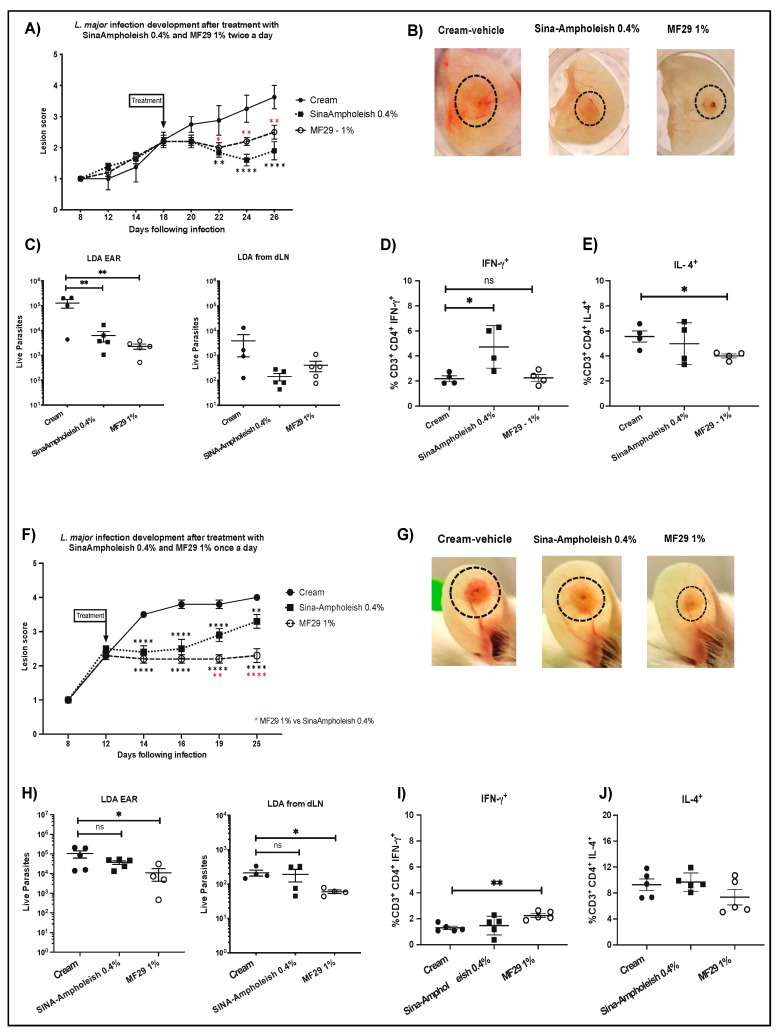
Comparison of MF29 and SinaAmpholeish topical treatments. BALB/c mice were infected i.d. with metacyclic *L. major* LV39-mCherry and lesion development was scored daily. Once the lesion reached a score of 2: (**A**) SinaAmpholeish 0.4%, MF29 1%, or cream vehicle were applied twice a day in each group of mice. Lesion score development was analyzed. (**B**) Representative pictures in each group at 26 days post-infection. (**C**) Twenty-six days post-infection, parasite load in the ear and dLN were analyzed by limiting dilution assay. (**D**) The frequency of CD4^+^IFN-γ^+^ and (**E**) CD4^+^IL-4^+^ production in dLN T cells was analyzed by flow cytometry. (**F**) Lesion development of mice treated once a day with MF29 1%, SinaAmpholeish 0.4% or vehicle cream. (**G**) Representative pictures of an infected ear at the end of the different treatments. (**H**) Parasite load from ear and dLN were determined by LDA. The IFN-γ^+^ (**I**) and IL-4 (**J**) production by CD4^+^ dLN T cells was analyzed by flow cytometry. For the infection data are represented as mean ± SEM, Two-Way ANOVA (Bonferroni’s post-hoc test) * *p* < 0.0332, ** *p* < 0.002, **** *p* < 0.0001, ns = not significant. For the LDA and cytokines, One-Way ANOVA non-parametric *T*-test (Dunn’s post-hoc) * *p* < 0.05, ** *p* < 0.002, ns = not significant. The data are representative of two experiments with 5 mice/group.

Recently, a new topical treatment for cutaneous leishmaniasis based on Amphotericin B (SinaAmpholeish) was developed [19]. We thus compared SinaAmpholeish 0.4%, which is recommended to be applied topically twice a day, with MF29 1% topical treatment. To this end, we infected BALB/c mice i.d. with *L. major* LV39 expressing mCherry. We followed lesion development and when the lesion reached a score of 2, treatments with either of the formulated creams were applied twice a day to the respective groups as previously reported here for MF29 1% and previously for SinaAmpholeish 0.4% [20]. Mice topically treated with MF29 1% and SinaAmpholeish 0.4% all showed smaller lesions compared with the control group (Figure 6A). Seven days post treatment (day 24 p.i.), the observed lesions were smaller in the SinaAmpholeish 0.4% treated mice post-treatment than in the MF29 1% treated mice, a difference that decreased nine days post treatment (day 26 p.i.) (Figure 6A black squares). Overall, cream-vehicle and MF29 1% infected ears showed less cutaneous inflammation than SinaAmpholeish 0.4% treated ones, but early signs of necrosis were observed at the end of the experiment in MF29 but not in SinAmpholeish treated ears (Figure 6B). Furthermore, the parasitic load at the infection site was lower in both MF29 1% and SinaAmpholeish 0.4% groups (Figure 6C) compared to the control group. A small but not significant difference was observed in the parasitic load observed in the dLN of mice treated with MF29 1% and SinaAmpholeish 0.4% compared to mice treated with vehicle cream (Figure 6C). The production of IFN-γ in CD4^+^ dLN T cells was significantly higher with approximately twice the frequency of cells positive for IFN-γ in the mice treated with the SinaAmpholeish 0.4% while again, no difference was observed in the frequency of CD4^+^IFNγ^+^ dLN cells between MF29 1% and vehicle control treated mice (Figure 6D). The results obtained with SinaApholeish 0.4% are in agreement with data previously reported for *L. mexicana* [20]. There was no difference in the frequency of IL-4 producing cells between the SinaAmpholeish 0.4% treated mice and control group (Figure 6E), but again, we observed that MF29 1% induced a significant reduction in the frequency of CD4^+^IL-4^+^ T cells (Figure 6E) compared to the control group.

Topical application of MF29 1% once a day had an impact on the outcome of the cutaneous lesion and parasite load control. We thus wanted to further investigate how this effect compared to topical treatment with SinaAmpholeish 0.4% applied once a day. We found that the protective effect of the SinaAmpholeish 0.4% observed when used twice daily (Figure 6A) was markedly decreased when the treatment was applied only once a day (Figure 6F). In contrast, MF29 1% treatment applied once a day was again shown to have a significant improvement in the outcome of disease (Figure 6F). The ear tissue showed necrosis at the end of the experiment in the control and SinaAmpholeish 0.4% groups, being larger in the cream-vehicle group, while the MF29 1% presented a slight sign of necrosis onset (Figure 6B). In addition, the parasite load in the MF29 1% group was significantly lower in the ear dermis and the dLN (Figure 6H) compared with the control group. In contrast, the parasitic load observed in the mice treated with SinaAmpholeish was similar to that observed in the control group (Figure 6H). The frequency of CD4^+^ T cells producing IFN-γ was slightly higher in mice treated with MF29 1% (Figure 6I). Of note, the increased expression of CD4^+^FNγ^+^ T cells observed was not always statistically significant when compared to the cream-vehicle group. In contrast, no difference was observed with the SinaAmpholeish 0.4% treated mice (Figure 6I) that had the same frequency of IFN-γ^+^ cells as the control group. In this experiment, we found no impact on the frequency of IL-4^+^ producing cells among the groups, but a small but not significant decrease in the frequency of CD4^+^IL-4^+^ CD4^+^ T cells in the MF29 1% treated group (Figure 6J). We thus show here that in contrast to SinaAmpholeish 0.4% topical treatment, which requires bi-daily application, MF29 1% topical cream application once daily is sufficient to significantly improve the disease outcome.

## 4. Discussion

Treatments against cutaneous leishmaniasis have been neglected, as most efforts have focused on visceral leishmaniasis, the deadly form of the disease. However, the morbidity associated with CL is very high and the development of new treatments is required. A general consensus is to use local treatment, including intralesional injection of sodium stibogluconate, heat treatment, cryotherapy, and topical treatment with Paromomycin or combinations of the afore mentioned treatments. Among these, topical formulations could be a rational approach for the cure of patients with few, localized or non-complicated lesions caused by *Leishmania* spp. from the Old and New World [21,22]. In this study, we investigated a potential topical use of MF29, a drug derived from 3-haloacetmidobenzoic acid. MF29 was previously reported to show an anti-*Leishmanial* effect in vitro and also in vivo when injected intra-peritoneally in *L. major* infected BALB/c mice [12]. We first observed parasite killing on in vitro promastigotes and on intracellular amastigotes in macrophages. The drug had in vitro leishmanicidal effects on both Old World *L. major* and New World *L. mexicana*, in line with a previous study [12]. In addition, we show here that the microbicidal effect of this drug was observed on two distinct *L. major* strains including the *L. major* Sd strain that was isolated from a non-healing lesion of a patient treated with multi courses of therapy, including pentavalent antimony and amphotericin B [23]. This *L. major* (Sd) strain causes the development of non-healing form of the disease in C57BL/6 mice [10], in contrast to the *L. major* (LV39), which, as with most of the commonly experimentally used *L. major* strains, induces the development of a small lesion that is self-healing [24].

We then aimed to test topical administration of MF29 at different concentrations and found that topical application of a MF29 1% formulation once a day, applied on an established BALB/c ear lesion, led to a significant improvement of the cutaneous disease progression already 12 days after topical application of the cream. The most significant impact was observed on the clear reduction of inflammation and on a decrease in parasite load observed both locally and in the dLN. Nevertheless, a small crust appeared around 20–25 days of treatment, which was significantly smaller than that observed in the ears of untreated BALB/c mice. Doubling the frequency of application to twice a day also resulted in improvement in the pathology but did not increase the benefit of the treatment compared to the treatment applied once a day. Most anti-leishmanial drugs are currently tested in BALB/c mice, often after a needle injection of a high dose of parasites injected subcutaneously in the hind footpad. Here we injected the parasites i.d. in the ear pinna, a mode of injection resembling more closely to the natural infection through infected sand fly bites. However, it is important to note that using either subcutaneous or intradermal injection, a high dose of parasites is inoculated, a process which is rarely observed following sand fly deposition of the parasites in the ear [25], but which allows rapid assessment of potential benefits of treatment.

BALB/c mice are highly susceptible to *L. major* infection, a well-established model correlating with the development of a strong Th2 immune response, characterized mostly by the presence of CD4^+^IL-4 secreting Th2 cells, low numbers of both IFN-γ and IL-10-secreting CD4^+^ T cells (reviewed in [7]). In contrast, infection of C57BL/6 mice with *L. major* induces a small lesion that is self-healing, a process linked with the differentiation of IFN-γ secreting Th1 cells and the absence of CD4^+^IL-4^+^ T cells [24]. We could show that topical treatment applied once a day when the lesion reached a score of 2, induced a significant improvement in BALB/c mice infected with *L. major* LV39. The most dramatic effect was the decreased inflammation observed following MF29 1% topical application, but of note, a small crust was often observed 25 days after infection, separating this process from that of inflammation. In addition, MF29 1% treatment decreased the Th2 immune response, as revealed by decreased IL-4 production and IgG1 levels in dLNs and sera, respectively, from *L. major* LV39 infected BALB/c mice. Thus, MF29 1% topical application results in increased control of cutaneous inflammation, parasite load, and a decreased Th2 immune response.

We also investigated the impact of the MF29 drug on *L. major* Sd, a *L. major* strain that causes a non-healing progressive lesion in C57BL/6 mice [10]. Following the infection of C57BL/6 mice with *L. major* Sd, no difference in IL-4 and IFN-γ production by dLN CD4^+^ T cells was observed between mice treated topically with vehicle or MF29 1% cream. IL-10 was reported to contribute to the pathology observed following infection with this strain of *L. major* [18]. A very small increase in CD4^+^IL-10^+^ producing cells was observed in dLN cells of mice topically treated with MF29 1%. However, the treatment did not influence the significant decrease in lesion pathology observed in MF29 1% treated mice. These results suggest that the impact of MF29 treatment on the control of the pathology following *L. major* Sd does not correlate with decreased IL-10 levels. The observed control of the inflammatory lesion by MF29 1% treatment involves distinct immune mechanisms than those observed following treatment of *L. major* LV39 infected BALB/c mice that included a decreased Th2 immune response. These differences are not surprising as distinct mechanisms are implicated in the pathology observed following infection with these two *L. major* species [10,26,27].

Most topical treatments currently tested are applied twice a day. These include Paromomycin topical treatment which is very cumbersome to produce [28,29]. Upon administration of Paromomycin twice a day during 20 days in a study performed in Tunisia, cure rates of 82% were observed, while vehicle control treatment gave a cure rate of 58%, revealing some improvement in this topical treatment [29]. Similar outcomes were observed for New World CL [30]. Attempts to topically apply Miltefosine were unsuccessful [31]. Recently, topical treatment based on Amphotericin B (SinaAmphoLeish), has been developed by the Drugs for Neglected Diseases initiative (DNDi); however, different outcomes have been reported depending on the *Leishmania* spp. used [20,32]. In the present investigation, we demonstrate that both MF29 1% and SinoAmpholeish 0.4% drugs are efficient against *L. major* LV39 when topically administrated twice a day, but the impact of each in the development of the immune response differ markedly, with the former decreasing IL-4 levels with a modest effect on IFN-γ levels, while SinaAmpholeish has a more pronounced impact on IFN-γ but none on IL-4 levels.

Most of the problems encountered with the efficacy of topical treatments may lie in the formulation of these drugs and their penetration rate [33]. Here we show that using experimental mouse models, topical application of MF29 1% once a day is sufficient to obtain a significant benefit on lesion development and parasite control.

Collectively, our results show that topical application of a MF29 1% formulated cream applied once a day has a significant beneficial effect on the pathology of a cutaneous lesion, making it a drug that warrants further research for promising cutaneous leishmaniasis applications.
